# Treatment discontinuation in a post‐disaster outpatient clinic: A retrospective cohort study of post‐traumatic stress disorder and complex post‐traumatic stress disorder in Fukushima, Japan

**DOI:** 10.1002/pcn5.70416

**Published:** 2026-09-23

**Authors:** Arinobu Hori, Emiko Ando, Naomi Ito, Michio Murakami, Masaharu Tsubokura

**Affiliations:** ^1^ Department of Psychiatry Hori Mental Clinic Minamisoma Fukushima Japan; ^2^ Department of Neuropsychiatry Fukushima Medical University School of Medicine Fukushima Fukushima Japan; ^3^ Department of Radiation Health Management Fukushima Medical University School of Medicine, Fukushima Fukushima Japan; ^4^ Center for Infectious Disease Education and Research (CiDER) The University of Osaka Suita Osaka Japan; ^5^ EIPM Center The University of Osaka Suita Osaka Japan

**Keywords:** complex PTSD, disaster psychiatry, post‐traumatic stress disorder (PTSD), staged treatment, treatment discontinuation

## Abstract

**Aim:**

Trauma‐focused psychotherapies are recommended as first‐line treatments for post‐traumatic stress disorder (PTSD); however, treatment discontinuation remains a major challenge in routine clinical practice. This study examined treatment discontinuation and structured psychotherapy implementation among patients with PTSD and complex PTSD (CPTSD) in a post‐disaster outpatient setting.

**Methods:**

This retrospective cohort study analyzed anonymized clinical records from April 2016 to March 2021 at a psychiatric outpatient clinic in Minamisoma City, Fukushima, Japan. Patients with ICD‐11 PTSD or CPTSD as a primary or secondary diagnosis were included (*n* = 170), with an analytic cohort (*n* = 144, excluding transfers, deaths, and undetermined outcomes) for structured psychotherapy analyses. To address immortal time bias arising from delayed trauma‐focused psychotherapy initiation (median 327 days from initial visit), structured psychotherapy was modeled as a time‐varying covariate in Cox regression.

**Results:**

Treatment discontinuation occurred in 33.5% (57/170) of patients within 1 year, with 64.9% of events occurring within the first 90 days. Structured psychotherapy was delivered to 17.6% (30/170) of patients. Although crude discontinuation rates differed markedly by trauma‐focused psychotherapy receipt (7.1% vs. 47.4%), the association was substantially attenuated and non‐significant in the time‐varying analysis (hazard ratio = 0.482, 95% CI 0.113–2.050), indicating that the crude association largely reflected immortal time bias.

**Conclusion:**

In this post‐disaster outpatient setting, discontinuation was concentrated in the early phase of care that precedes structured psychotherapy, and most patients never reached trauma‐focused treatment. Early treatment engagement, rather than structured psychotherapy delivery alone, may represent the critical bottleneck in real‐world PTSD care.

## INTRODUCTION

Post‐traumatic stress disorder (PTSD) is a common psychiatric condition that often follows a prolonged and chronic course in the absence of appropriate intervention. Core symptoms include intrusive recollections, avoidance of trauma‐related cues, negative alterations in cognition and mood, and heightened arousal, all of which are associated with significant impairment in social and occupational functioning^1^, ^2^.

Over the past decades, trauma‐focused psychotherapies—including prolonged exposure therapy (PE), cognitive processing therapy (CPT), and eye movement desensitization and reprocessing (EMDR)—have been established as first‐line treatments for PTSD, supported by substantial evidence from randomized controlled trials^3^, ^4^, ^5^, ^6^, ^7^. However, the implementation of these treatments in routine clinical practice remains challenging. A substantial proportion of patients discontinue treatment prematurely or fail to initiate trauma‐focused interventions, raising concerns about the real‐world effectiveness of evidence‐based therapies.

Previous studies have documented high rates of treatment discontinuation in PTSD care^8^, ^9^. However, most evidence has been derived from clinical trials or administrative datasets, and relatively little is known about how treatment discontinuation unfolds over time within routine outpatient settings. In particular, the temporal dynamics of disengagement—such as whether discontinuation occurs before or after initiation of structured psychotherapy—remain insufficiently characterized. Understanding when patients disengage from treatment may be critical for identifying modifiable barriers to sustained care^10^.

In routine clinical practice, treatment for trauma‐related disorders typically follows a phased process. After initial assessment, patients often undergo a period of stabilization and engagement, during which acute symptoms are managed and a therapeutic alliance is established. Trauma‐focused psychotherapies are usually introduced after this preparatory phase. This sequential structure implies that patients must remain engaged in care for a sufficient duration before accessing evidence‐based psychotherapies. Consequently, early disengagement from treatment may represent a critical barrier that limits access to effective interventions.

The issue of treatment engagement may be particularly salient in disaster‐affected regions, where long‐term psychological distress, disrupted social infrastructure, and limited healthcare resources can complicate the delivery of mental health services^11^, ^12^. Following the Great East Japan Earthquake and the subsequent nuclear accident in Fukushima in 2011, residents in affected areas have experienced persistent psychological challenges. Previous reports from psychiatric services in this region have highlighted both the difficulty of providing adequate trauma‐focused care in routine settings and the feasibility of such interventions when patients remain engaged^13^, ^14^, ^15^, ^16^, ^17^. However, these studies did not examine the temporal dynamics of treatment discontinuation or the point at which patients disengage from care.

The introduction of complex PTSD (CPTSD) in the ICD‐11 classification has emphasized the heterogeneity of trauma‐related disorders. In addition to core PTSD symptoms, CPTSD is characterized by disturbances in self‐organization (DSO), including affect dysregulation, negative self‐concept, and interpersonal difficulties^18^, ^19^. These features may influence patterns of treatment engagement and discontinuation within the clinical pathway, although empirical evidence in routine clinical settings remains limited.

Against this background, the present study aimed to examine treatment discontinuation and structured psychotherapy implementation in a real‐world outpatient clinic in a post‐disaster setting. Using 5 years of retrospective clinical data, we evaluated 1‐year treatment discontinuation rates and conducted survival analyses to characterize the timing of disengagement. In particular, the study focused on whether discontinuation occurred before patients reached structured psychotherapy, thereby examining potential vulnerabilities within the treatment pathway rather than the effectiveness of trauma‐focused psychotherapy itself.

## METHODS

### Study design and setting

This retrospective cohort study was conducted at a psychiatric outpatient clinic in Minamisoma City, Fukushima Prefecture, Japan, an area affected by the Great East Japan Earthquake and subsequent nuclear disaster in 2011. The clinic was established in April 2016, following the 2011 disaster, and provides routine psychiatric care, including pharmacotherapy and psychotherapy, for a wide range of mental health conditions, with a particular focus on trauma‐related disorders. In addition to routine brief outpatient visits, structured psychotherapy sessions were provided to patients at no extra cost, supported by funding from People's Hope Japan, a non‐profit organization. This arrangement allowed for a level of trauma‐focused psychotherapy provision that exceeds what is typically available in standard Japanese outpatient psychiatric settings. Clinical data were extracted from anonymized medical records covering the clinic's full operational period from April 2016 to March 2021.

### Participants

All patients who made an initial visit to the clinic during the study period (*N* = 2234) were screened for inclusion. The study population consisted of patients who met diagnostic criteria for post‐traumatic stress disorder (PTSD) or complex post‐traumatic stress disorder (CPTSD) according to ICD‐11 concepts (codes 6B40 and 6B41, respectively). Both primary and secondary diagnoses were reviewed, and patients were included if PTSD or CPTSD was identified as either the primary or secondary diagnosis. Diagnoses were based on the treating psychiatrist's clinical assessment; in some cases, the diagnosis was established over the course of several visits or was revised during follow‐up as the clinical picture became clearer. This yielded an ICD‐11 PTSD/CPTSD subgroup of 170 patients (PTSD *n* = 81; CPTSD *n* = 89). The remaining 2,064 patients were used only for descriptive comparison of baseline characteristics and 1‐year outcomes (Table 1).

**Table 1:** Patient characteristics: ICD‐11 PTSD/CPTSD subgroup versus all other patients.

Characteristic	PTSD/CPTSD subgroup (*n* = 170)	Other patients (*n* = 2064)	*p*
Demographics			
Age, mean ± SD (years)	41.2 ± 20.5	49.1 ± 25.6	<0.001
Male sex, *n* (%)	55 (32.4)	852 (41.3)	0.023
Treatment characteristics			
Structured psychotherapy within observation period, *n* (%)	30 (17.6)	15 (0.7)	<0.001
One‐year outcome, *n* (%)			
Remission with planned termination	6 (3.5)	179 (8.7)	
Ongoing treatment at 1 year	81 (47.6)	975 (47.2)	
Transfer to another facility	14 (8.2)	160 (7.8)	
Treatment discontinuation	57 (33.5)	548 (26.6)	
Death	2 (1.2)	17 (0.8)	
Other/undetermined	10 (5.9)	185 (9.0)	0.080

*Note*: The ICD‐11 PTSD/CPTSD subgroup comprises patients with post‐traumatic stress disorder (ICD‐11 code 6B40) or complex post‐traumatic stress disorder (6B41) as either primary or secondary diagnosis. Age: independent samples *t*‐test; sex and structured psychotherapy: Fisher's exact test; 1‐year outcome distribution: chi‐square test (χ^2^ = 9.832, df = 5, *p* = 0.080; one cell had an expected count less than 5 [death category]). Abbreviations: CPTSD, complex post‐traumatic stress disorder; PTSD, post‐traumatic stress disorder; SD, standard deviation.

**Figure 1:**
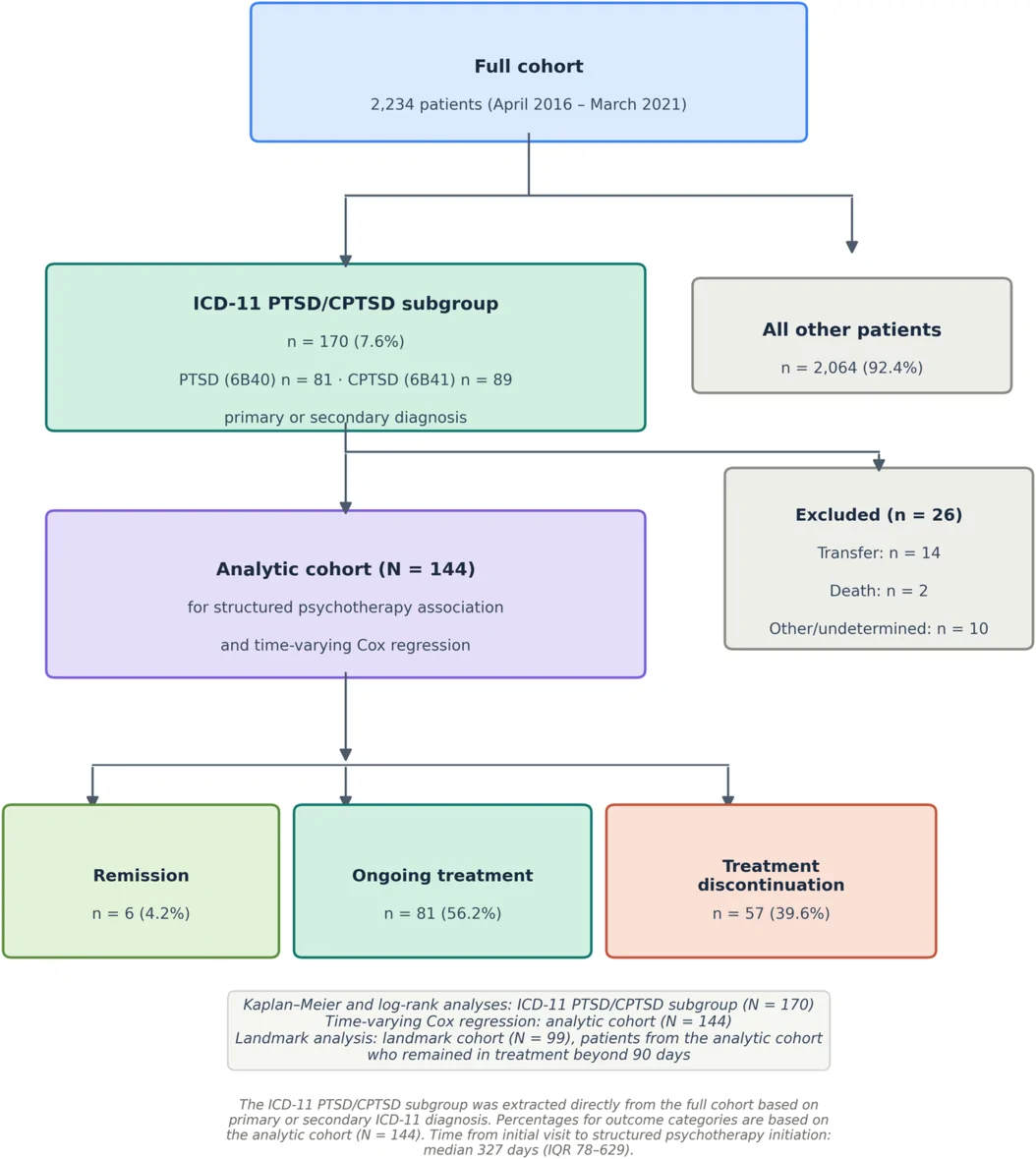
Study flowchart showing patient selection and 1‐year outcomes.

For the analyses of structured psychotherapy and treatment discontinuation, a restricted subset was defined, referred to as the analytic cohort (*N* = 144). This cohort was constructed by excluding patients whose 1‐year outcome was classified as transfer to another facility (*n* = 14), death (*n* = 2), or other/undetermined (*n* = 10), as these outcomes reflect neither sustained treatment engagement nor clinically meaningful discontinuation. The analytic cohort therefore comprised patients with outcomes of remission with planned termination (*n* = 6), ongoing treatment at 1 year (*n* = 81), or treatment discontinuation (*n* = 57). The relationships among these groups are summarized in Figure 1.

### Diagnostic classification

Diagnoses were made in routine clinical practice by the treating psychiatrist (the first author), who has extensive clinical experience in trauma‐related disorders and holds certification in Prolonged Exposure therapy and Schema Therapy (Advanced Level, Individual). Diagnostic assessments were based on clinical interviews and were consistent with ICD‐11 diagnostic concepts. Patients were assigned to the PTSD (6B40) or CPTSD (6B41) subgroups based on whether either diagnosis appeared as a primary or secondary diagnosis.

### Outcome definition

#### Treatment discontinuation (binary outcome)

For the primary analysis, treatment discontinuation within 1 year of the initial visit was defined as cessation of outpatient visits within 12 months, without documented clinical improvement, planned termination, transfer to another facility, or death, and without subsequent re‐attendance at the clinic by March 2024. This definition was designed to reflect clinically meaningful discontinuation rather than administrative loss to follow‐up and to minimize misclassification of patients who temporarily interrupted treatment.

Discontinuation status was independently confirmed by two clinicians, including the treating psychiatrist.

#### Time to discontinuation (survival outcome)

For survival analysis, time to treatment discontinuation was calculated as the number of days from the initial visit to the last recorded outpatient visit. Patients who continued treatment beyond 1 year or remained in treatment at the end of the observation period were treated as censored cases.

Survival analysis was employed to account for variability in follow‐up duration across patients and to allow examination of patterns of early versus late disengagement from treatment over time.

The proportion of discontinuation events occurring within the first 90 days was calculated based on event counts from the survival data.

#### Structured psychotherapy

Structured psychotherapy was defined as the provision of structured psychological interventions with a trauma‐focused component, delivered by a psychiatrist or a clinical psychologist. Prolonged exposure therapy^7^ was the primary modality; other interventions incorporating trauma‐focused elements were also included. Sessions were conducted within a dedicated appointment of approximately 60 min (or 90 min in the case of prolonged exposure therapy); brief therapeutic interactions during routine short outpatient visits were not included. Patients who did not receive structured psychotherapy nevertheless received routine outpatient psychiatric care, which in Japanese practice typically includes brief supportive psychotherapeutic interactions, psychoeducation, crisis management, and pharmacotherapy delivered within regular outpatient visits.

Because structured psychotherapy was typically initiated only after a period of clinical stabilization, the interval from initial visit to structured psychotherapy commencement varied considerably among patients who received it (median 327 days, IQR 78–629 days). Trauma‐focused psychotherapy was considered appropriate for patients who presented with clear PTSD re‐experiencing symptoms (such as flashbacks and nightmares) and demonstrated sufficient motivation and willingness to engage with trauma‐related material. Patients were excluded from structured psychotherapy if significant contraindications were present, including active suicidal ideation, severe depressive or manic episodes, or alcohol or substance dependence. The decision to initiate trauma‐focused psychotherapy was made through collaborative discussion between the treating psychiatrist and the patient. The resulting variation in the timing of structured psychotherapy relative to treatment duration has important analytic implications. Classifying structured psychotherapy as a fixed baseline exposure would introduce immortal time bias; the strategy used to address this is described in the Statistical analysis section.

### Statistical analysis

#### Analysis of 1‐year treatment discontinuation

Baseline characteristics and 1‐year outcomes were compared between the ICD‐11 PTSD/CPTSD subgroup and all other patients. Continuous variables (age) were compared using independent samples *t*‐tests, categorical variables (sex and structured psychotherapy receipt) using Fisher's exact test, and the overall distribution of 1‐year outcomes using the chi‐square test. For descriptive purposes, odds ratios (ORs) and 95% confidence intervals (CIs) for the association between structured psychotherapy and treatment continuation were calculated within the analytic cohort using a fixed classification of structured psychotherapy receipt; as detailed below, this fixed classification is vulnerable to immortal time bias and is reported for descriptive comparison only.

#### Survival analysis

Kaplan–Meier survival analysis was used to estimate treatment continuation probabilities in the ICD‐11 PTSD/CPTSD subgroup (*N* = 170), with differences between diagnostic groups assessed by the log‐rank test. Treatment discontinuation was the event of interest; remission with planned termination, transfer, death, and ongoing treatment at the end of observation were censored.

Because structured psychotherapy was typically initiated only after an extended period of assessment and stabilization (median 327 days from the initial visit), classifying structured psychotherapy as a fixed baseline exposure would introduce immortal time bias: patients who eventually received structured psychotherapy necessarily remained in treatment long enough to reach initiation and could not, by definition, have discontinued beforehand. To address this, the primary analysis modeled structured psychotherapy as a time‐varying covariate in a Cox proportional hazards regression within the analytic cohort (*N* = 144). For each patient who initiated structured psychotherapy during the observation period, the covariate was coded as 0 from the initial visit until the date of structured psychotherapy initiation and as 1 thereafter; for all other patients, it remained 0 throughout. Diagnosis (CPTSD vs. PTSD), age, and sex were included as covariates. A conventional Cox model treating structured psychotherapy as a fixed baseline exposure was also computed and is reported for comparison, explicitly labeled as vulnerable to immortal time bias.

As a complementary description of early‐discontinuation dynamics, a landmark analysis was conducted, restricted to patients in the analytic cohort who remained in treatment for more than 90 days from the initial visit (landmark cohort, *N* = 99), with time zero set at the 90‐day landmark. This landmark was pre‐specified based on the observation that 64.9% of discontinuation events occurred within the first 90 days. Because the median time to structured psychotherapy initiation (327 days) exceeded the landmark, a 90‐day landmark alone does not fully remove immortal time bias for structured psychotherapy exposure; the time‐varying analysis therefore constitutes the primary approach, and the landmark analysis is presented as a complementary description.

#### Additional analyses

Descriptive analyses were conducted to examine yearly trends in treatment discontinuation and structured psychotherapy implementation rates. These analyses were intended to assess whether changes in clinical practice over time were associated with changes in overall treatment retention at the facility level.

All statistical analyses were conducted using Python (version 3.12.3; Python Software Foundation). Survival analysis, including Kaplan–Meier estimation, log‐rank tests, and Cox proportional hazards regression, was performed using the lifelines library (version 0.30.3). Fisher's exact test and Student's *t*‐test were conducted using the scipy.stats module (SciPy version 1.17.0). A two‐sided *p*‐value of <0.05 was considered statistically significant.

## RESULTS

### Study population

During the study period, 2234 patients attended the outpatient clinic. Of these, 170 patients (7.6%) met ICD‐11 criteria for PTSD (*n* = 81) or CPTSD (*n* = 89) as a primary or secondary diagnosis; the remaining 2064 patients formed the comparison group. The patient selection process is summarized in Figure 1.

Compared with the other patients, those in the ICD‐11 PTSD/CPTSD subgroup were younger (mean age 41.2 ± 20.5 vs. 49.1 ± 25.6 years, *p* < 0.001) and less likely to be male (32.4% vs. 41.3%, *p* = 0.023). Structured psychotherapy was delivered to 17.6% (30/170) of the PTSD/CPTSD subgroup, compared with 0.7% (15/2064) of other patients (*p* < 0.001). Detailed comparisons are presented in Table 1.

Patient characteristics of the ICD‐11 PTSD and CPTSD subgroups are presented in Table 2.

**Table 2:** Patient characteristics of the ICD‐11 PTSD/CPTSD subgroup (*N* = 170).

	PTSD (*n* = 81)	CPTSD (*n* = 89)	*p*
Demographics			
Age, years (mean ± SD)	48.4 ± 21.6	34.6 ± 17.0	<0.001
Female sex, *n* (%)	49 (60.5)	66 (74.2)	0.071
Treatment characteristics			
Observation duration, days (mean ± SD)	205.2 ± 165.4	241.2 ± 156.2	0.146
Structured psychotherapy received, *n* (%)	14 (17.3)	16 (18.0)	1.000
One‐year outcome, *n* (%)			
Remission with planned termination	4 (4.9)	2 (2.2)	–
Ongoing treatment at 1 year	34 (42.0)	47 (52.8)	–
Transfer to another facility	5 (6.2)	9 (10.1)	–
Treatment discontinuation	32 (39.5)	25 (28.1)	–
Death	1 (1.2)	1 (1.1)	–
Other/undetermined	5 (6.2)	5 (5.6)	–

*Note*: *p* values: age and observation duration, independent samples *t*‐test; sex and structured psychotherapy, Fisher's exact test. The overall 1‐year outcome distribution was not formally tested between PTSD and CPTSD subgroups because five of 12 cells had an expected count less than 5, violating the assumptions of the chi‐square test. Abbreviations: CPTSD, complex post‐traumatic stress disorder; PTSD, post‐traumatic stress disorder; SD, standard deviation.

### One‐year treatment outcomes

At 1 year, treatment discontinuation was observed in 33.5% (57/170) of the PTSD/CPTSD subgroup, compared with 26.6% (548/2,064) of other patients. Patients in the PTSD/CPTSD subgroup were less likely to achieve remission with planned termination (3.5% vs. 8.7%). The overall distribution of 1‐year outcomes did not differ significantly between groups (χ^2^ = 9.832, df = 5, *p* = 0.080; one cell had an expected count less than 5). Detailed distributions are presented in Table 1.

### Structured psychotherapy and treatment discontinuation

Among the 170 patients in the ICD‐11 PTSD/CPTSD subgroup, 30 received structured psychotherapy during the observation period. Prolonged exposure therapy was the most common modality (*n* = 14, 46.7%); other modalities included EMDR (*n* = 3), schema therapy (*n* = 2), and other or unspecified trauma‐focused interventions (*n* = 11). Of these 30 patients, 28 were included in the analytic cohort (*N* = 144); the remaining two were excluded because their 1‐year outcome was classified as transfer or other/undetermined. Under a fixed classification of structured psychotherapy receipt, treatment discontinuation occurred in 7.1% (2/28) of patients who received structured psychotherapy and 47.4% (55/116) of those who did not (odds ratio for treatment continuation = 9.57, 95% CI 2.49–36.77, *p* < 0.001). The full 1‐year outcome distribution by structured psychotherapy status is presented in Table 3; patients who received structured psychotherapy were more likely both to remain in ongoing treatment (82.1% vs. 50.0%) and to achieve remission with planned termination (10.7% vs. 2.6%).

**Table 3:** One‐year outcomes by structured psychotherapy receipt (analytic cohort, *N* = 144).

	Structured psychotherapy (*n* = 28)	No structured psychotherapy (*n* = 116)
One‐year outcome, *n* (%)		
Remission with planned termination	3 (10.7)	3 (2.6)
Ongoing treatment at 1 year	23 (82.1)	58 (50.0)
Treatment discontinuation	2 (7.1)	55 (47.4)
Descriptive association		
Odds ratio for treatment continuation (95% CI)	9.57 (2.49–36.77)	Reference
Fisher's exact test	*p* < 0.001	

*Note*: The analytic cohort was derived from the ICD‐11 PTSD/CPTSD subgroup (*N* = 170) by excluding patients with transfer to another facility (*n* = 14), death (*n* = 2), or other/undetermined outcome (*n* = 10). The odds ratio classifies structured psychotherapy as a fixed binary exposure and is presented for descriptive purposes only; this classification is vulnerable to immortal time bias, because patients had to remain in treatment to initiate structured psychotherapy (median time to initiation: 327 days). The primary, time‐varying analysis of structured psychotherapy and treatment continuation, which accounts for this bias, is presented in Table 5. Odds ratio calculated with Haldane–Anscombe correction. Abbreviations: CI, confidence interval; CPTSD, complex post‐traumatic stress disorder; PTSD, post‐traumatic stress disorder.

This fixed‐classification comparison, however, is vulnerable to immortal time bias, because the median time from initial visit to structured psychotherapy initiation was 327 days (IQR 78–629); most patients who received structured psychotherapy had therefore already been in treatment for an extended period before initiation. In the primary analysis modeling structured psychotherapy as a time‐varying covariate, the association was substantially attenuated and no longer statistically significant (HR = 0.482, 95% CI 0.113–2.050, *p* = 0.323). Diagnosis, age, and sex were likewise not significantly associated with treatment discontinuation (Table 5). For comparison, the conventional fixed‐exposure Cox model yielded an HR of 0.099 (95% CI 0.024–0.406, *p* = 0.001), illustrating the magnitude of the bias introduced by ignoring exposure timing.

### Fiscal year trends

In the analytic cohort (*N* = 144), overall discontinuation rates varied across fiscal years without a consistent temporal trend. Structured psychotherapy implementation declined markedly in fiscal year 2020, coinciding with the onset of the COVID‐19 pandemic in Japan. Detailed year‐by‐year patterns are presented in Table 4.

**Table 4:** Fiscal year trends in treatment discontinuation and structured psychotherapy implementation (analytic cohort, *N* = 144).

Fiscal year	*n*	Overall discontinuation *n*/*N* (%)	PT *n*	Discontinuation by structured psychotherapy status	
				No structured psychotherapy *n*/*N* (%)	Structured psychotherapy *n*/*N* (%)
2016	47	18/47 (38.3)	7	18/40 (45.0)	0/7 (0.0)
2017	27	12/27 (44.4)	6	11/21 (52.4)	1/6 (16.7)
2018	15	2/15 (13.3)	5	2/10 (20.0)	0/5 (0.0)
2019	39	18/39 (46.2)	9	17/30 (56.7)	1/9 (11.1)
2020	16	7/16 (43.8)	1	7/15 (46.7)	0/1 (0.0)

*Note*: Fiscal year runs from April to March (e.g., fiscal year 2016 = April 2016 to March 2017). PT, structured psychotherapy. Overall discontinuation rate is calculated for all patients in the analytic cohort per fiscal year. Discontinuation by structured psychotherapy status shows the number and proportion of patients who discontinued treatment within each subgroup. The analytic cohort (*N* = 144) is derived from the ICD‐11 PTSD/CPTSD subgroup (*N* = 170), excluding patients with transfer, death, or undetermined outcomes.

### Survival analysis

**Figure 2:**
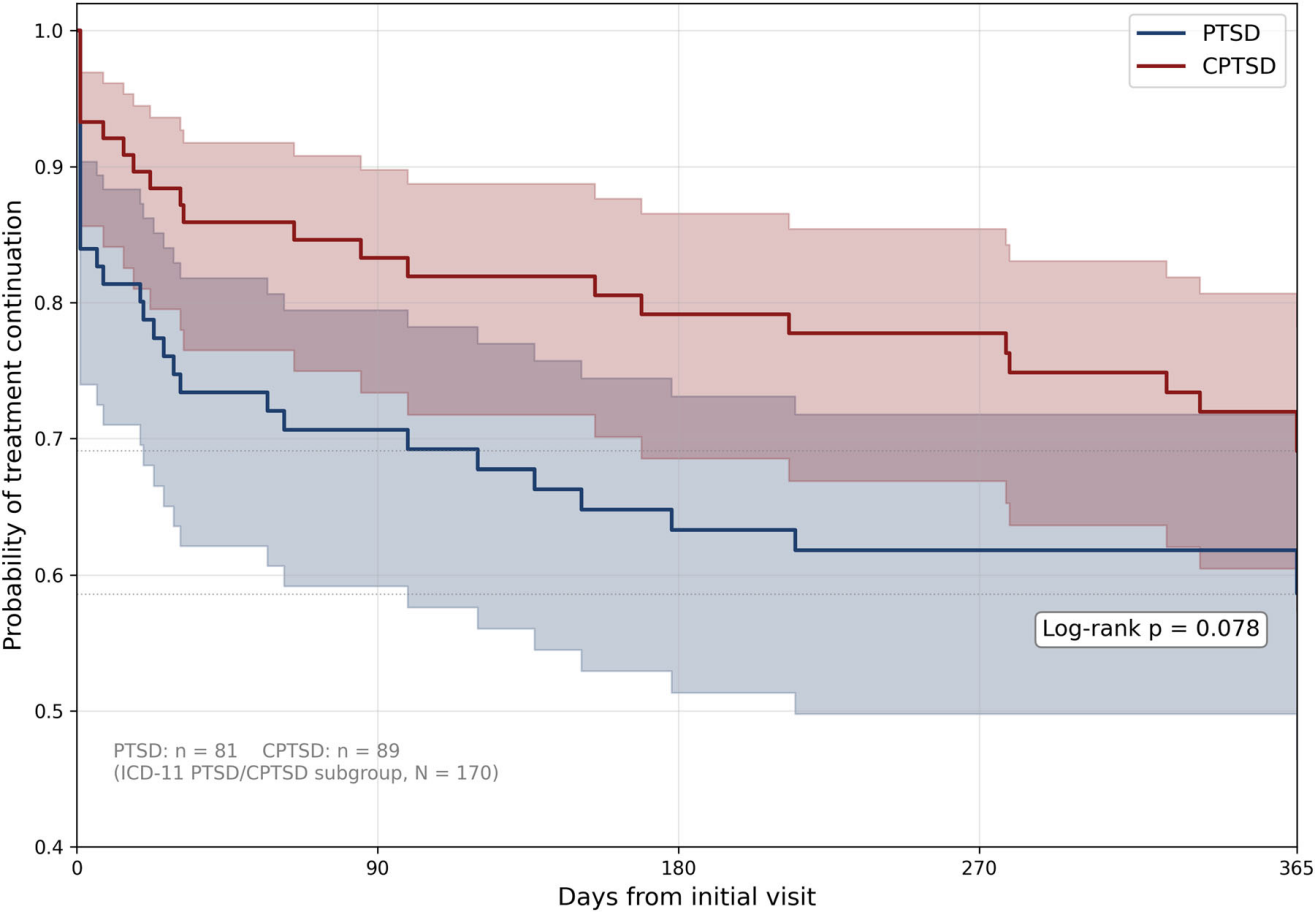
Kaplan–Meier curves for probability of treatment continuation in the ICD‐11 PTSD/CPTSD subgroup (*N* = 170), stratified by diagnosis (PTSD vs. complex PTSD). Log‐rank *p* = 0.078. Shaded areas indicate 95% confidence intervals. 64.9% of discontinuation events occurred within the first 90 days.

Kaplan–Meier analysis in the ICD‐11 PTSD/CPTSD subgroup (*N* = 170) showed 1‐year treatment continuation probabilities of 58.6% for PTSD and 69.1% for CPTSD (log‐rank *p* = 0.078; Figure 2). The decline in treatment continuation was most pronounced within the first 90 days, during which 64.9% of all discontinuation events occurred. Time‐point continuation probabilities are presented in Table 5.

**Table 5:** Survival analysis results: Kaplan–Meier treatment continuation probabilities and Cox proportional hazards regression.

A. Treatment continuation probability by time point				
Time point	Full ICD‐11 PTSD/CPTSD subgroup (*N* = 170)		Landmark cohort (*N* = 99)	
	PTSD (*n* = 81)	CPTSD (*n* = 89)	PTSD (*n* = 44)	CPTSD (*n* = 55)
Kaplan–Meier treatment continuation probability				
90 days	70.6%	83.3%	–	–
180 days	63.3%	79.1%	88.6%	94.5%
270 days	61.8%	77.7%	86.4%	92.7%
365 days	58.6%	69.1%	81.7%	81.8%
Log‐rank test (PTSD vs. CPTSD)	*p* = 0.078		*p* = 0.806	
*Note*: The landmark cohort includes patients who remained in treatment beyond 90 days (*N* = 99). Continuation probabilities for the landmark cohort are calculated from the 90‐day landmark; “–” indicates the time point precedes the landmark. Log‐rank test compares PTSD and CPTSD within each cohort.				

B. Cox proportional hazards regression						
	Analytic cohort (*n* = 144, events = 57)			Landmark cohort (*n* = 99, events = 20)		
**Covariate**	**HR**	**95% CI**	***p***	**HR**	**95% CI**	***p***
Time‐varying Cox regression (primary analysis)						
Structured psychotherapy(time‐varying)	0.482	0.113–2.050	0.323	–	–	–
Diagnosis (CPTSD vs. PTSD)	0.620	0.353–1.088	0.096	–	–	–
Age (per year)	0.996	0.982–1.010	0.608	–	–	–
Sex (male vs. female)	1.459	0.849–2.507	0.171	–	–	–
Fixed‐exposure Cox regression (for comparison; vulnerable to immortal time bias)						
Structured psychotherapy (fixed)	0.099	0.024–0.406	0.001	0.266	0.061–1.158	0.078
Diagnosis (CPTSD vs. PTSD)	0.630	0.357–1.113	0.111	0.824	0.318–2.138	0.691
Age (per year)	0.993	0.980–1.007	0.342	0.999	0.978–1.022	0.961
Sex (male vs. female)	1.540	0.896–2.647	0.118	0.475	0.137–1.652	0.242
Concordance index (fixed‐exposure model): analytic cohort = 0.695; landmark cohort = 0.636						

*Note*: The primary analysis modeled structured psychotherapy as a time‐varying covariate (coded 0 before structured psychotherapy initiation and 1 thereafter) to avoid immortal time bias. The fixed‐exposure model, which classifies structured psychotherapy as a baseline characteristic, is reported for comparison and substantially overestimates the association because patients had to remain in treatment (median 327 days) to initiate structured psychotherapy. The time‐varying model was not applied to the landmark cohort, for which the fixed‐exposure estimate is shown. HR < 1 indicates a lower hazard of treatment discontinuation (i.e., improved continuation).

Abbreviations: CI, confidence interval; CPTSD, complex post‐traumatic stress disorder; HR, hazard ratio; PTSD, post‐traumatic stress disorder.

### Landmark analysis at 90 days

**Figure 3:**
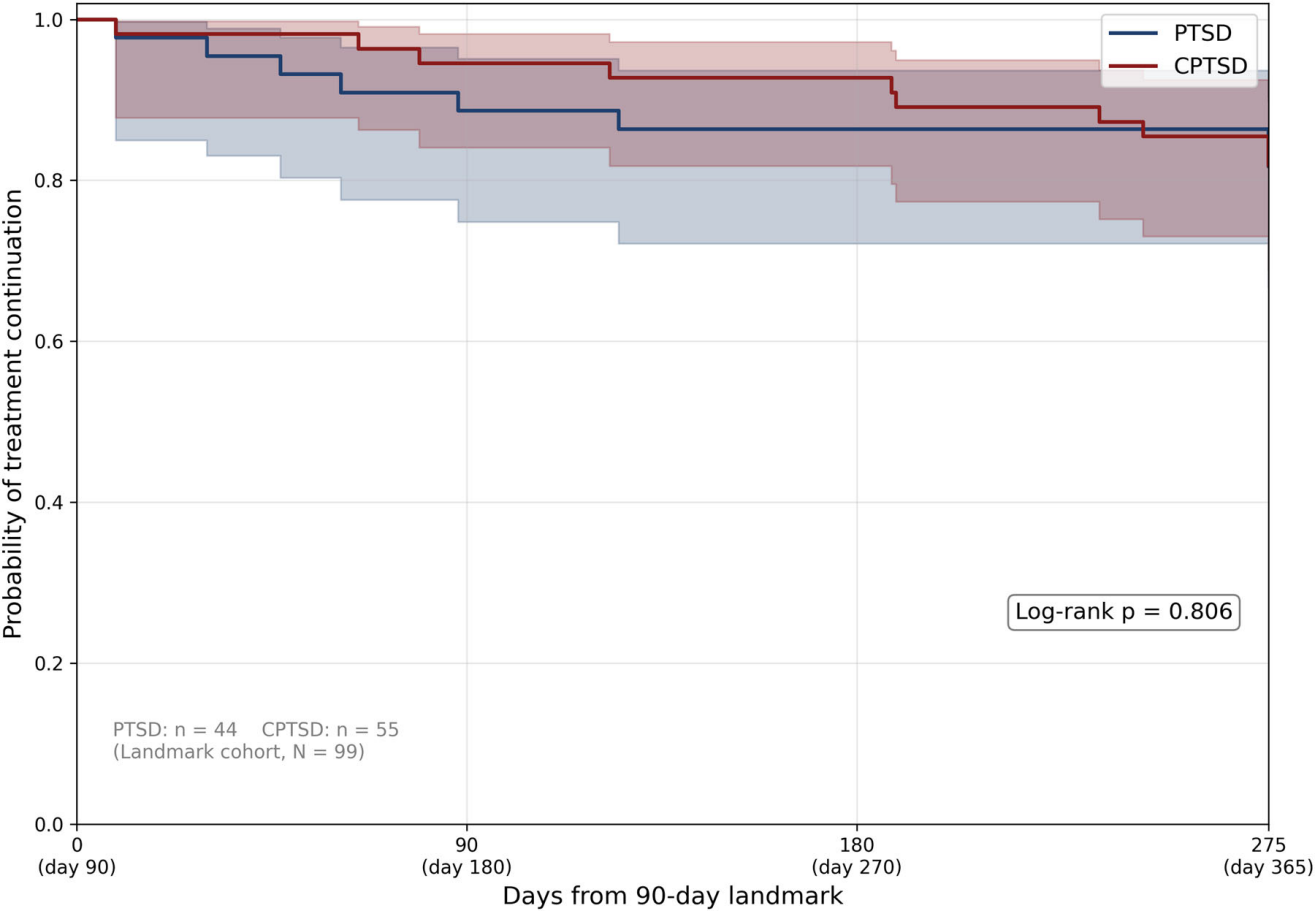
Kaplan–Meier curves for treatment continuation probability in the landmark cohort (*N* = 99), restricted to patients who remained in treatment beyond 90 days from the initial visit. Time zero represents the 90‐day landmark. Log‐rank *p* = 0.806. Shaded areas indicate 95% confidence intervals.

Among the 144 patients in the analytic cohort, 99 (68.8%) remained in treatment beyond 90 days and were included in the landmark analysis (PTSD *n* = 44, CPTSD *n* = 55). In this cohort, 20 discontinuation events were observed, corresponding to substantially higher continuation probabilities than in the full subgroup: 81.7% for PTSD and 81.8% for CPTSD at 1 year (log‐rank *p* = 0.806; Figure 3). Once patients remained engaged beyond the initial 90‐day period, the subsequent risk of discontinuation was low and did not differ by diagnosis (Table 5).

## DISCUSSION

This retrospective cohort study examined treatment discontinuation among patients with ICD‐11 PTSD and CPTSD in a post‐disaster outpatient psychiatric clinic in Fukushima, Japan. Three main findings emerged. First, treatment discontinuation within 1 year was common, affecting 33.5% of patients in the PTSD/CPTSD subgroup, and was heavily concentrated in the early phase of care: 64.9% of all discontinuation events occurred within the first 90 days. Second, structured trauma‐focused psychotherapy was delivered to only 17.6% of patients, and its initiation was typically late (median 327 days from the initial visit). Third, although patients who received structured psychotherapy showed markedly lower crude discontinuation rates, this association was substantially attenuated and no longer significant when structured psychotherapy was modeled as a time‐varying exposure (HR = 0.482, 95% CI 0.113–2.050), indicating that the crude association largely reflected immortal time bias rather than a protective effect of structured psychotherapy itself.

In routine clinical practice, trauma‐focused psychotherapies are typically introduced only after an initial phase of assessment, stabilization, and engagement, during which a therapeutic alliance is established and acute symptoms are addressed. The concentration of discontinuation within the first 90 days therefore indicates that many patients did not remain in care long enough to transition into this later phase of treatment. From this perspective, the central challenge identified in this study is not limited to the effectiveness of trauma‐focused psychotherapy itself, but a structural vulnerability in the treatment pathway: disengagement occurs before patients are able to reach the interventions regarded as first‐line treatment. Dropout from PTSD treatment is a well‐documented phenomenon, with meta‐analytic estimates indicating that a substantial proportion of patients discontinue before completing evidence‐based care^8^, ^9^; our findings extend these observations by locating the highest‐risk period in the early phase that precedes structured psychotherapy.

The contrast between the fixed‐exposure and time‐varying analyses is itself instructive. When structured psychotherapy receipt was treated as a fixed baseline characteristic, it appeared strongly protective (odds ratio for continuation = 9.57; fixed‐exposure HR = 0.099). Modeling structured psychotherapy as a time‐varying covariate—coding each patient as unexposed until the actual date of initiation—attenuated the hazard ratio approximately fivefold and rendered it non‐significant. This pattern is a characteristic signature of immortal time bias: because the median time to structured psychotherapy initiation was 327 days, patients who received structured psychotherapy had, by definition, already remained in treatment long enough to reach it. We present both estimates deliberately, as the magnitude of the discrepancy may be instructive for other researchers analyzing structured psychotherapy delivery in routine‐care cohorts, where delayed treatment initiation is common. Importantly, these findings do not indicate that structured psychotherapy is ineffective; rather, they indicate that the present observational data cannot demonstrate an effect on retention, and that patients who remained in care long enough to initiate structured psychotherapy were already a selected group at low risk of discontinuation. The wide confidence interval of the time‐varying estimate, reflecting only 28 exposed patients, also precludes ruling out a clinically meaningful effect.

The landmark analysis reinforces the centrality of the early phase. Among patients who remained in treatment beyond 90 days, 1‐year continuation probabilities exceeded 80% in both diagnostic groups (PTSD 81.7%, CPTSD 81.8%), and no variable—including structured psychotherapy—significantly predicted subsequent discontinuation. Once patients passed the initial high‐risk window, retention appeared largely secure regardless of diagnosis. Because the median time to structured psychotherapy initiation exceeded the 90‐day landmark, this analysis does not isolate an effect of structured psychotherapy; rather, together with the time‐varying analysis, it converges on a single interpretation—that the critical determinant of treatment failure is retention through the early phase, not the subsequent delivery of structured psychotherapy to already‐engaged patients.

At the population level, only 17.6% of the PTSD/CPTSD subgroup received structured psychotherapy within the observation period, despite the availability of trauma‐focused sessions at no additional cost. Importantly, patients who did not receive structured psychotherapy were not untreated: they received routine outpatient psychiatric care, which in Japanese practice includes brief supportive psychotherapeutic contact, psychoeducation, and pharmacotherapy within regular visits. The low implementation rate of structured psychotherapy therefore reflects the combined effect of early discontinuation, clinical eligibility criteria, patient readiness, and the capacity constraints of a small post‐disaster clinic, rather than an absence of therapeutic contact. Trauma‐focused psychotherapies such as prolonged exposure therapy, cognitive processing therapy, and EMDR have demonstrated efficacy in controlled trials^5^, ^6^, yet their real‐world impact depends on patients remaining engaged long enough to access them; similarly low engagement has been observed in large healthcare systems, where only a minority of patients with PTSD initiate or complete evidence‐based psychotherapy^20^. Structured psychotherapy implementation declined further in fiscal year 2020, coinciding with the COVID‐19 pandemic, suggesting that public health crises can compound existing barriers to structured psychotherapy delivery in post‐disaster settings.

The concept of “access” to care is often invoked to describe such gaps; however, the present findings more specifically reflect challenges in treatment engagement within a defined clinical pathway. The study did not include direct measures of factors that may influence access or healthcare utilization, such as geographical distance, socioeconomic status, family support, or transportation availability. Alternative explanations, including individual symptom profiles, motivational factors, or contextual barriers, may also have influenced treatment trajectories. These considerations underscore the need for future studies incorporating more comprehensive assessments of the determinants of engagement, particularly in disaster‐affected settings where structural and social factors may play a substantial role^11^, ^12^.

Differences in treatment continuation between PTSD and CPTSD were modest and did not reach statistical significance in survival analysis. Although continuation probabilities were numerically higher among patients with CPTSD, these findings should be interpreted cautiously given the absence of statistical significance and the exploratory nature of subgroup comparisons. At the same time, the observed pattern may still be clinically meaningful. One possible interpretation is that patients with PTSD, whose symptom presentation is often dominated by trauma‐related avoidance, may be more vulnerable to early disengagement within the treatment process when treatment begins to approach traumatic experiences. In contrast, patients with CPTSD may, despite substantial avoidance, remain engaged for longer. This may reflect the therapeutic process itself, including being listened to with sustained clinical interest and having broader emotional and interpersonal difficulties recognized. These factors may help address important unmet emotional needs. Although this interpretation remains speculative and cannot be tested directly in the present dataset, it may offer a clinically relevant hypothesis for understanding differential engagement patterns across trauma‐related disorders. This possibility is broadly consistent with prior work highlighting the heterogeneity of trauma‐related disorders and the complex interplay between symptom profiles, relational needs, and treatment engagement^18^, ^19^.

From a clinical perspective, these findings highlight the importance of interventions targeting early‐phase engagement in treatment. Given that a substantial proportion of patients disengage before reaching structured psychotherapy, efforts to improve outcomes may need to focus on the initial stages of care. Potential strategies include more structured follow‐up during the early treatment period, proactive outreach, and interventions aimed at enhancing motivation and readiness for treatment. Low‐intensity or preparatory interventions may also serve as a bridge to more intensive trauma‐focused therapies. In this context, staged treatment approaches have been proposed as a means of facilitating engagement prior to trauma processing, with emerging evidence suggesting potential benefits in complex PTSD populations^21^. Within this framework, preparatory interventions such as Skills Training in Affective and Interpersonal Regulation (STAIR;^22^) and schema therapy^23^, ^24^ may represent promising intermediate steps, helping patients develop emotional regulation and gain insight into maladaptive coping patterns prior to engaging with traumatic memories. A longitudinal case report from the same clinical setting described a staged approach—progressing from routine outpatient care through schema therapy to prolonged exposure—that appeared effective in a patient with delayed‐onset PTSD following the 2011 disaster^25^. Further empirical investigation is needed to establish whether such staged approaches can improve early treatment retention in routine post‐disaster care.

Taken together, the findings of this study suggest that treatment discontinuation in routine post‐disaster psychiatric care is best understood as a problem of early disengagement within the clinical pathway, rather than as a failure of treatment efficacy. Addressing this early‐phase vulnerability may be critical for improving the real‐world effectiveness of PTSD care and ensuring that patients are able to reach evidence‐based psychotherapies. These findings extend previous observations from this clinical setting^13^, ^17^ and are consistent with reports from other settings^26^, ^27^. Future studies incorporating symptom‐level and process‐level measures may help clarify the determinants of early disengagement.

## LIMITATIONS

Several limitations should be considered when interpreting the findings of this study.

First, this was a retrospective observational study conducted at a single outpatient clinic, which may limit the generalizability of the findings to other clinical settings and healthcare systems. The unique context of a post‐disaster region and the availability of externally funded trauma‐focused psychotherapy may further constrain external validity.

Second, although the time‐varying analysis addresses immortal time bias, it cannot address confounding by indication: patients selected for structured psychotherapy were required to have sufficient stability and motivation, characteristics that independently predict retention. The association between structured psychotherapy and continuation should therefore be interpreted as descriptive of the clinical pathway rather than as a causal effect.

Third, the study did not include direct measures of factors that may influence treatment access or healthcare utilization, such as geographical distance from the clinic, socioeconomic status, transportation availability, family support, or occupational constraints. Consequently, the mechanisms underlying early treatment disengagement cannot be directly determined, and the observed patterns may be attributable to a combination of clinical, social, and contextual factors that were not captured in the dataset.

Fourth, diagnostic classification was based on routine clinical assessment rather than structured diagnostic interviews, which may have introduced variability in diagnostic accuracy. Although diagnoses were made by an experienced clinician using ICD‐11‐informed concepts, misclassification cannot be excluded.

Fifth, although treatment discontinuation was carefully defined, some patients classified as having discontinued treatment may have continued care at other institutions. Such transitions could not be systematically captured, and therefore discontinuation rates may be overestimated.

Finally, potentially important clinical variables—including symptom severity, comorbid psychiatric conditions, medication regimens, and patient motivation or readiness—were not systematically assessed. These factors may influence both treatment engagement and the likelihood of receiving structured psychotherapy. In particular, pharmacotherapy, delivered to most patients as part of routine care, was not analyzed and may itself affect stabilization and retention. The small number of patients who initiated structured psychotherapy during the observation period (*n* = 28) also limits the precision of the time‐varying estimate.

## CONCLUSIONS

In this post‐disaster outpatient setting, treatment discontinuation was common, early, and concentrated in the phase of care that precedes structured psychotherapy. Most patients never reached trauma‐focused treatment, and the apparent protective association of structured psychotherapy with retention was largely attributable to immortal time bias. These findings redirect attention from trauma‐focused psychotherapy delivery to early treatment engagement as the critical bottleneck in real‐world PTSD care. Interventions targeting the first 90 days of care—structured follow‐up, proactive outreach, and preparatory or low‐intensity interventions—may hold greater potential for reducing overall treatment failure than expanding structured psychotherapy capacity alone. Further research is needed to clarify the determinants of early disengagement and to identify effective approaches for improving early retention in routine clinical settings.

## AUTHOR CONTRIBUTIONS

A.H. conceived and designed the study, collected and analyzed the data, and drafted the manuscript. E.A. contributed to data entry from clinical records, assisted with outcome verification, and critically reviewed the manuscript. M.T. and M.M. contributed to critical review and revision of the manuscript. N.I. assisted with ethics approval procedures and contributed to critical review and revision of the manuscript. All authors read and approved the final version of the manuscript.

## CONFLICT OF INTEREST STATEMENT

The authors declare no conflicts of interest.

## ETHICS APPROVAL STATEMENT

This study was conducted in accordance with the ethical standards of the Declaration of Helsinki. The study protocol was approved by the Ethics Committees of Minamisoma City General Hospital (Number: 1–12) and Fukushima Medical University (Number: 2019‐271). All data were handled in anonymized form.

## PATIENT CONSENT STATEMENT

Individual patient consent was waived by the ethics committees as the study used anonymized retrospective clinical data. All data were handled in accordance with applicable privacy regulations.

## CLINICAL TRIAL REGISTRATION

This study is a retrospective observational study and was not registered as a clinical trial.

## Data Availability

The data that support the findings of this study are not publicly available due to patient privacy and ethical restrictions. Anonymized data may be made available from the corresponding author upon reasonable request and subject to ethics committee approval.
